# Weight Loss, Pathological Changes, and Inflammatory Effects from a Short-Term Ketogenic Diet in Overweight and Obese Men with Untreated Prostate Cancer on Active Surveillance

**DOI:** 10.3390/nu16213716

**Published:** 2024-10-30

**Authors:** Adeel Kaiser, Mohummad M. Siddiqui, Jason Bosley-Smith, Shu Wang, Joseph Aryankalayil, Mark V. Mishra, Alice S. Ryan, Christopher R. D’Adamo

**Affiliations:** 1 Department of Radiation Oncology, University of Maryland School of Medicine, Baltimore, MD 21201, USA; joseph.b.aryankalayil.mil@health.mil (J.A.); mmishra@umm.edu (M.V.M.); 2 Department of Radiation Oncology, Miami Cancer Institute, Miami, FL 33176, USA; 3 Herbert Wertheim College of Medicine, Florida International University, Miami, FL 33199, USA; 4 Division of Urology, Department of Surgery, University of Maryland School of Medicine, Baltimore, MD 21201, USA; msiddiqui@som.umaryland.edu (M.M.S.); sh.wang@som.umaryland.edu (S.W.); 5 Center for Integrative Medicine, University of Maryland School of Medicine, Baltimore, MD 21201, USA; jbosley13@gmail.com; 6 Department of Medicine, University of Maryland School of Medicine, Baltimore, MD 21201, USA; aryan@som.umaryland.edu; 7 Department of Family and Community Medicine, University of Maryland School of Medicine, Baltimore, MD 21201, USA; cdadamo@som.umaryland.edu

**Keywords:** obesity, prostate cancer, active surveillance, ketogenic diet, overweight, inflammation

## Abstract

Background and Aims: Active Surveillance (AS) is a favored strategy for the management of indolent prostate cancers (PCs). Overweight and obese men harbor an increased risk of cancer progression during AS. We aim to prospectively evaluate the feasibility and outcomes of a ketogenic diet (KD) weight-loss intervention in overweight men with PC. Materials and Methods: Men with PC and a BMI > 25 kg/m^2^ undergoing AS were placed on an 8-week ad libitum KD program before a scheduled surveillance biopsy to assess the impact on clinical grade group (CGG). Blood ketone levels were tracked to ensure compliance. BMI, PSA, and inflammatory marker data (TNF-α, TNFR1, TNFR2, sICAM-1, sVCAM-1, IL-6, IL1-RA, CRP, and SAA) were collected before and after the KD intervention. A Shapiro–Wilk test was performed to assess the normality of all continuous study variables. Paired t-tests and Wilcoxon rank sum tests were utilized to compare normally and non-normally distributed study outcomes, respectively. Results: Ten AS patients aged 62.1 (±5.4) years were enrolled with an average BMI of 31.7 kg/m^2^ (±11.8). Post-KD intervention mean blood ketone levels were 0.32 (±0.12) mmol/L with a mean BMI reduction of 7.4% (*p* < 0.0003). There were no meaningful changes in PSA or inflammatory biomarkers (*p* > 0.05). Nine patients completed re-biopsy following a KD with four patients showing no evidence of cancer; one downgraded to a lower CGG; two had unchanged CGG scores; and two had higher CGG scores compared to baseline. Conclusions: Short-term KD interventions for BMI reduction are feasible in men undergoing AS for PC and may result in favorable pathological effects without inflammatory marker changes. Larger studies with longer follow-up are needed to explore whether KD-induced weight loss can improve clinical outcomes with AS in PC.

## 1. Introduction

Almost 300,000 men are diagnosed with prostate cancer (PC) each year, with over 35,000 men succumbing to the disease annually [1]. Although radiation and prostatectomy offer excellent cure rates for men with localized PC, these treatments are often accompanied by gastrointestinal, urinary, and sexual toxicities that can significantly impact quality of life [2,3]. To avoid these issues, many men with lower grade forms of PC select active surveillance (AS) instead of treatment. This approach entails prostate-specific antigen (PSA) level measurement at 3–6 month intervals with surveillance prostate biopsies for periodic reassessment or if indicated by serial PSA elevations [4,5]. Treatments are only completed if surveillance biopsy results confirm progression to more aggressive PC. Ideally, invasive procedures such as surgery and radiotherapy could be delayed indefinitely with this strategy.

Unfortunately, available data suggests that up to 55% of men on AS will ultimately require treatment for PC due to progression [6,7]. Overweight and obesity status has been identified as a major risk factor for AS failure. In a Canadian study of 565 men undergoing AS for PC, a 50% increased risk of progression was observed for every 5 kg/m^2^ increase in BMI over 25 [8]. Thus, it is hypothesized that weight loss may provide substantial clinical benefit for overweight men with PC on AS.

A low-carbohydrate, ketogenic diet (KD) approach has been proposed as a clinically beneficial strategy to limit PC progression by mitigating negative factors, including metabolic syndrome, insulin resistance, and hormonal therapies [9]. In this study, we examine the feasibility and outcomes of an 8-week KD intervention in men who are overweight and obese undergoing AS. Given the relationship between obesity, proinflammatory factors (e.g., IL-6, TNFR2, and CRP), and prostate cancer progression, we also examined the effect of a KD on markers of inflammation [10,11]. A short-term intervention period was also selected for safety because of conflicting preclinical data regarding high-fat diet-induced prostate cancer progression via adipokines in certain animal models [12].

## 2. Materials and Methods

### 2.1. Enrollment

Following approval by the Institutional Research Board of the University of Maryland School of Medicine, a pilot, single-arm clinical trial was initiated examining the feasibility and efficacy of an 8-week KD intervention for overweight men (BMI ≥ 25 kg/m^2^) undergoing AS for PC. The clinical trial was registered at ClinicalTrials.gov (NCT03194516). All recruited patients were diagnosed with PC and met guidelines for AS with a Gleason score ≤ 7, clinical stage T1c–T2a, PSA < 10 ng/mL, and survival expectancy of ≥ 10 years [13]. Participants were required to have an Eastern Cancer Oncology Group performance status ≤2 and a life expectancy > 1 year. Patients with diabetes, steroid medication, and/or prior or concurrent anti-androgen deprivation therapy were excluded since these factors may impact glucose levels, ketosis, and body weight, and could not be stratified for due to the smaller sample size intended for this pilot study.

### 2.2. Intervention

Study participants received in-person or virtual one-on-one training from a licensed dietitian to implement the KD strategy, including grocery shopping and food preparation instructions. This included a pre-intervention discussion as well as weekly phone calls during the study period. Phone calls were meant to ensure dietary compliance as well as to gather ketone level data from each subject. The eight-week KD intervention included a 3:1 ratio of fat grams to the grams supplied by protein and carbohydrate to achieve ketosis. Participants were instructed to eat to satiety without a defined caloric limit. They were also provided a digital scale and glucose/ketone meter with strips to measure and record blood glucose and ketone levels at weekly intervals. They were instructed to avoid any changes in baseline exercise activity. The start date for the KD intervention for each patient was timed such that the two-month diet intervention would conclude on the day of a scheduled MRI-guided surveillance prostate biopsy. The re-biopsy was meant to provide preliminary data regarding pathological changes with a KD as assessed by scoring changes in the clinical grade group (CGG).

For participants starting with a more standard/conventional dietary macronutrient ratio composition (IOM-calculated acceptable macronutrient distribution range = carbohydrate (CHO) (45–65% of energy), protein (PRO) (10–35% of energy), and fat (20–35% of energy), a gradual reduction in CHO with a simultaneous gradual increase in fat was employed to ensure greater compliance and mitigate potential adverse events. Adjustment to the ketogenic diet was handled on an individual basis by the licensed dietician on the study with the aim of achieving ketosis within one week of diet initiation. Dietary intake was then monitored by the study dietitian throughout the intervention, and compliance was assessed by blood ketone levels, which were checked on a weekly basis. Generally, fat intake increases were advised with foods containing high proportions of medium-chain triglycerides due to their uniquely favorable properties related to digestion and absorption. Participant education and support packets were provided to supplement one-on-one dietary instruction. High-fat foods, including full-fat dairy, fatty fish, meats, nuts, and seeds, were recommended. Avocado, coconut, butter, and palm kernel oils were advised for cooking.

### 2.3. Outcomes

The primary outcomes included feasibility (as expressed by completion of the intervention and blood ketone levels at the conclusion of the intervention) and efficacy of the 8-week KD intervention in reducing baseline BMI. A 5% reduction in mean BMI was deemed clinically meaningful and plausible based on previously reported weight loss with ad libitum ketogenic diets in obese individuals [14]. Secondary outcomes included PSA, pathology results from re-biopsy, and plasma biomarkers of inflammation. Fasting plasma for C-reactive protein (CRP), acute-phase serum amyloid A (SAA), intracellular adhesion molecule (sICAM-1), and vascular CAM-1 (sVCAM-1) was measured in duplicate, and tumor necrosis factor alpha (TNF-α), TNF receptor 1 (TNFR1), TNF receptor 2 (TNFR2), interleukin-6 (IL-6), and interleukin-1 receptor antagonist (IL1-RA) were measured in triplicate with a CV less than 10% according to electrochemiluminescence using a multi-spot microplate (SECTOR Imager-2400, Meso Scale Discovery, Gaithersburg, MD, USA).

### 2.4. Statistical Analysis

Descriptive statistics (means, standard deviations, frequencies, etc.) were computed to characterize the study sample. A Shapiro–Wilk test was performed to assess the normality of all continuous study variables. Paired *t*-tests were utilized to compare normally distributed study outcomes, and Wilcoxon rank sum tests were employed to compare non-normally distributed outcomes before and after the ketogenic diet intervention. Pearson correlation coefficients were also calculated to estimate the correlations between weight and the plasma biomarkers under study before and after the intervention. Statistical significance was defined as *p* < 0.05. All statistical analyses were performed in SAS Version 9.4.1 (Cary, NC, USA).

## 3. Results

Ten participants enrolled in the clinical trial. The mean (SD) age of the participants was 62.1 (±5.4) years. Subjects self-identified as Caucasian (60%), African American (20%), and other (20%). All ten participants (100%) completed the ketogenic diet intervention. Mean (SD) blood ketone levels were 0.32 (0.12) mmol/L at the conclusion of the intervention. Only one subject did not achieve a 0.3 mmol/L ketone level with a KD. However, this patient still maintained ketone levels between 0.2 and 0.27 mmol/L during the entire study period.

Table 1 provides a comparison of pre-/post-intervention outcomes for weight, BMI, glucose, PSA, and inflammatory factors. In brief, there were statistically and clinically significant reductions in mean weight and BMI (7.4% reduction, *p* < 0.0003). However, there were no meaningful changes in PSA or any of the inflammatory biomarkers (*p* > 0.05). Mean glucose levels also remained unchanged from week 1 to week 8 of the intervention (93.3 ± 16.8 vs. 98.2 ± 10.8, *p* = 0.42). There were positive correlations between weight at baseline and CRP (r = 0.85, *p* = 0.004) and SAA (r = 0.89, *p* = 0.001) but no other meaningful correlations at baseline or the end of the study (*p* > 0.05).

Nine patients completed surveillance re-biopsy on the last day of the KD intervention with one patient declining the procedure. Re-biopsy results showed no evidence of cancer in four patients, downgraded cancer in one patient (CGG 2 to 1), and two patients with unchanged tumor grades from baseline. Tumor progression was seen in two patients. One subject progressed from CGG1 to 2 with a corresponding PSA rise from 7.2 to 8.8 ng/mL. This was despite a 12.1% reduction in BMI with a KD. The second patient progressed from CGG1 to 3 even though his PSA reduced from 10.4 to 9.6 ng/mL while his BMI decreased by 4.6%.

## 4. Discussion

Numerous guidelines now recommend that AS should be offered as a primary management strategy for patients with low-risk PC, with recent data indicating that 60% of eligible men select this approach [13,15]. However, as many as 55% of men will clinically progress with rising PSA and aggressive pathological changes within 10 years of starting AS [6,7]. It has been well-established that obesity is associated with cancer development and progression among many malignancies. Being overweight significantly increases the risk of both PC recurrence and progression [10]. Furthermore, as low-risk PC has a very low expected cancer specific mortality [3], a weight-loss intervention motivated by the new cancer diagnosis may provide an opportunity for a global improvement in health that is most meaningful for the patient’s overall mortality.

Our data showed that PSA levels remained stable with a short-term KD. Although longer duration data is needed to validate the clinical significance of this approach, these results are reassuring since PSA testing remains the primary mode of prostate cancer assessment during AS [5]. Pathology results were also encouraging with half the cohort experiencing clinical remission or tumor downgrading in comparison to two patients exhibiting progression by re-biopsy. Given the small study cohort, these preliminary results should be viewed as exploratory only.

Although low-carbohydrate diet interventions have been examined in the setting of post-treatment failure after primary therapy for prostate cancer [16], our study represents the first reported study of a KD in untreated patients undergoing AS to avoid primary PC therapies such as prostatectomy and radiation. A KD is intriguing due to recent reports indicating that ketones function as histone deacetylase inhibitors (HDACi), which are being explored in multidrug antitumor regimens for various cancers and may be useful in PC [17]. Additionally, metabolomic analysis of men with PC on low-carbohydrate diets suggests a potential link between ketogenesis and TCA metabolite profiles associated with slower PC growth [18]. Therefore, a KD may mechanistically alter disease progression in manners distinct from caloric restriction, exercise, or other weight-loss approaches. 

Several inflammatory indices (IL-6, TNFR2, and CRP) are positively associated with prostate cancer progression [11]. CRP and SAA are major acute phase proteins and are reduced following weight loss [19,20], with about a 0.13 mg/L reduction in CRP for every 1 kg of weight lost. This effect on SAA and CRP was not reflected in our data, possibly due to the limited size of the study cohort. In a prospective study of obese patients, a 12-month weight reduction program involving diet, exercise, and behavioral counseling resulted in 10% weight loss as well as statistically significant reductions in TNF-α, IL-6, ICAM-1, and VCAM-1 [21]. We did not observe reductions in those markers, potentially due to the shorter duration of our study intervention, exclusion of diabetic patients with more severe proinflammatory states at baseline, and/or our smaller patient sample size. Additionally, unlike other weight loss approaches, available data suggests that a KD may not alter TNFα despite moderate to large effects on body weight [22].

The feasibility of the KD intervention in this population was manifested in study participants successfully concluding the intervention with mean ketone concentrations beyond 0.3 mmol/l, which are considered clinically meaningful levels [23]. While larger studies with longer follow-up are needed to confirm the findings from this small pilot study, data from this study may attenuate potential concerns from preclinical studies that the very-high-fat content of a KD may have a deleterious impact on markers of inflammation in an acute setting.

## 5. Conclusions

In summary, overweight and obese men harbor an increased risk of clinical progression while undergoing AS for PC. Short-term KD interventions are a feasible option to help mitigate this risk without deleterious increases in inflammatory markers. Given current obesity rates and the reality that PC represents 30% of all cancers diagnosed each year in men [1], the potential impact of this preliminary data should not be overlooked. Larger, longer prospective studies are needed to assess whether weight loss using a KD may improve clinical outcomes for men on active surveillance for prostate cancer. These studies should also compare a KD with other dietary regimens to determine the best weight-loss strategy for this patient population.

## Figures and Tables

**Table 1:** Study outcomes pre- and post-ketogenic diet intervention.

Outcome	Pre-Intervention	Post-Intervention	*p* Value
Weight (lbs) *	236.3 (79.9)	221.2 (75.6)	0.0003
BMI (kg/m^2^) *	34.2 (11.8)	31.7 (10.8)	0.0003
Glucose (mg/dL) *	93.3 (16.8)	98.2 (10.8)	0.42
PSA (ng/mL) *	6.2 (3.5)	6.2 (3.1)	0.47
TNF-α (pg/mL) *	12.1 (2.4)	12.0 (2.8)	0.83
TNFR1 (ng/mL) *	2.3 (0.9)	2.0 (0.5)	0.71
TNFR2 (ng/mL) *	4.8 (1.5)	4.6 (1.4)	0.70
sICAM-1 (ng/mL)	311.7 (48.8)	289.2 (41.2)	0.09
sVCAM-1 (ng/mL)	382.7 (98.7)	392.1 (117.2)	0.42
IL-6 (pg/mL) **	2.1 (0.9–11.1)	2.1 (1.1–23.0)	0.76
IL1-RA (pg/mL) **	242.8 (170.2–915.2)	207.4 (131.2–381.7)	0.36
CRP (ng/mL) **	1242.6 (242.3–10,774.0)	2060.2 (306.1–3516.4)	0.68
SAA (ng/mL) **	3518.3 (1792.3–49,237.5)	2487.5 (1326–9065.8)	0.60

* Mean (SD) and *p* value calculated by paired t-test for normally distributed outcomes. ** Median (interquartile range) and *p* value calculated by Wilcoxon rank sum test for non-normally distributed outcomes; tumor necrosis factor alpha (TNF-α); TNF receptor 1 (TNFR1); TNF receptor 2 (TNFR2); intracellular adhesion molecule (sICAM-1); vascular CAM-1 (sVCAM-1); interleukin-6 (IL-6); interleukin-1 receptor antagonist (IL1-RA); C-reactive protein (CRP); acute-phase serum amyloid A (SAA).

## Data Availability

The original contributions presented in the study are included in the article, further inquiries can be directed to the corresponding author.

## Abbreviations

KD	Ketogenic diet
AS	Active surveillance
PC	Prostate cancer
